# Application of a deep learning image classifier for identification of Amazonian fishes

**DOI:** 10.1002/ece3.9987

**Published:** 2023-05-01

**Authors:** Alexander J. Robillard, Michael G. Trizna, Morgan Ruiz‐Tafur, Edgard Leonardo Dávila Panduro, C. David de Santana, Alexander E. White, Rebecca B. Dikow, Jessica L. Deichmann

**Affiliations:** ^1^ Data Science Lab Office of the Chief Information Officer, Smithsonian Institution Washington District of Columbia USA; ^2^ Center for Conservation and Sustainability Smithsonian National Zoo and Conservation Biology Institute Washington District of Columbia USA; ^3^ Chesapeake Biological Laboratory University of Maryland Center for Environmental Science Solomons Maryland USA; ^4^ Laboratorio de Taxonomía de Peces Instituto de Investigaciones de la Amazonía Peruana (IIAP) San Juan Bautista Peru; ^5^ Division of Fishes, Department of Vertebrate Zoology, MRC 159, National Museum of Natural History Smithsonian Institution Washington District of Columbia USA; ^6^ Working Land and Seascapes, Conservation Commons Smithsonian Institution Washington District of Columbia USA

**Keywords:** computer vision, conservation technology, deep machine learning, freshwater fish, Neotropical, species identification

## Abstract

Given the sharp increase in agricultural and infrastructure development and the paucity of widespread data available to support conservation management decisions, a more rapid and accurate tool for identifying fish fauna in the world's largest freshwater ecosystem, the Amazon, is needed. Current strategies for identification of freshwater fishes require high levels of training and taxonomic expertise for morphological identification or genetic testing for species recognition at a molecular level. To overcome these challenges, we built an image masking model (U‐Net) and a convolutional neural net (CNN) to classify Amazonian fish in photographs. Fish used to generate training data were collected and photographed in tributaries in seasonally flooded forests of the upper Morona River valley in Loreto, Peru in 2018 and 2019. Species identifications in the training images (*n* = 3068) were verified by expert ichthyologists. These images were supplemented with photographs taken of additional Amazonian fish specimens housed in the ichthyological collection of the Smithsonian's National Museum of Natural History. We generated a CNN model that identified 33 genera of fishes with a mean accuracy of 97.9%. Wider availability of accurate freshwater fish image recognition tools, such as the one described here, will enable fishermen, local communities, and citizen scientists to more effectively participate in collecting and sharing data from their territories to inform policy and management decisions that impact them directly.

## Graphical abstract

To address the growing prevalence of large ecological datasets combined with a need for rapid species identification tools, we describe a novel application of a computer vision model, which is built to rapidly identify Amazonian fishes from captured image data. We describe using a unique combination of field and museum‐sourced images to generate our training data, from which a validation subset was used to ensure model efficacy.

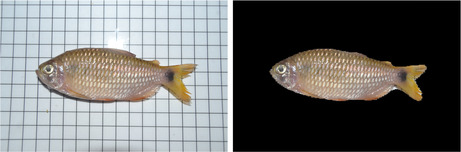

## Resumen

Dado el aumento del desarrollo agrícola e infraestructura y la escasa información disponible para apoyar la toma de decisiones con respecto al manejo y la conservación de la fauna, es necesario contar con una herramienta más rápida y precisa para la identificación de peces en el ecosistema de agua dulce más grande del mundo, la Amazonía. Las estrategias actuales para la identificación de peces de agua dulce requieren altos niveles de capacitación y experiencia taxonómica para la identificación morfológica o las pruebas genéticas para el reconocimiento de especies a nivel molecular. Para superar estos desafíos, construimos un modelo de enmascaramiento de imágenes (U‐Net) y una red neuronal convolucional (CNN) para clasificar los peces amazónicos en las fotografías. Los peces utilizados para generar datos de entrenamiento fueron recolectados y fotografiados en afluentes de bosques inundables de la cuenca alta del río Morona en Loreto, Perú en 2018 y 2019. Las identificaciones de especies en las imágenes de entrenamiento (*n* = 3.068) fueron verificadas por ictiólogos expertos. Estas imágenes se complementaron con fotografías tomadas de ejemplares adicionales de peces amazónicos alojados en la colección ictiológica del Museo Nacional de Historia Natural del Smithsonian en Washington, DC. Se generó un modelo CNN que identificó 33 géneros de peces con una precisión media del 97,9%. Una mayor disponibilidad de herramientas precisas de reconocimiento de imágenes de peces de agua dulce, como la que se describe aquí, permitirá a los pescadores, las comunidades amazónicas y los “científicos ciudadanos” participar de manera más efectiva en la recopilación y el intercambio de datos de sus territorios para informar las políticas y decisiones de gestión que los afectan directamente.

## 1 INTRODUCTION

The Amazon basin is home to over 2700 species of freshwater fishes (Dagosta & De Pinna, 2019; Junk et al., 2007), many of which are of conservation concern (Albert et al., 2011; García‐Dávila et al., 2018; Pelicice et al., 2021). Freshwater fishes provide one of the few reliable sources of protein for Amazonian communities and represent an important economic opportunity through the aquarium trade (Coomes et al., 2010; Moreau & Coomes, 2007). This unique ichthyofauna is facing unprecedented threats, such as deforestation (Junk et al., 2007; Lobón‐Cerviá et al., 2015), construction of hydropower dams (Winemiller et al., 2016), mining (Azevedo‐Santos et al., 2021), climate change (Bodmer et al., 2017), and in some cases, over exploitation (Moreau & Coomes, 2007). While advances in sampling poorly explored areas and describing the diversity of Amazonian fish have been made over the last decade (e.g., Alofs et al., 2014; de Santana et al., 2019, 2021), the sub‐drainages of the Marañón river remain among the most under sampled regions in South America (Jézéquel et al., 2020). Freshwater fishes provide one of the few reliable sources of protein for Amazonian communities (Coomes et al., 2010; Moreau & Coomes, 2007). In less populated areas of the Amazon, subsistence fishing, for both consumption and the pet trade, can be essential to sustaining life (Coomes et al., 2010; Moreau & Coomes, 2007). Due to the urgency of these economic and ecological threats, efficient data collection and long‐term monitoring are needed to better inform mitigation strategies and policy.

Traditional ichthyological sampling methods include focused netting and fishing efforts, followed by extensive manual sorting, documentation, and identification. Although effective, and necessary in the Amazon where a countless number of fishes remain to be described (Reis et al., 2016), these methods are time consuming and raise the potential for misidentification bias (Kirsch et al., 2018). As a result, many have turned to the assistance of community scientists to aid in catch effort and identification of individual landings, yet accurate species identification remains a challenge (Gardiner et al., 2012; Swanson et al., 2016). Genetic approaches have also been implemented to identify many of the fish species inhabiting the Amazon (de Santana et al., 2021; García‐Dávila et al., 2017), but these approaches also rely on well‐identified and vouchered genetic libraries that are still missing for Amazonian fishes. These techniques require expensive storage and sample processing technology, which are not readily available in most institutions within the Amazon (de Santana et al., 2021). In order to address the ever‐growing need for data and cost‐effective solutions, contemporary fisheries research has called for the development and application of a rapid solution, namely by way of machine learning models, such as Convolutional Neural Networks (CNNs, e.g., Perdigão et al., 2020). CNNs have the potential to enable rapid identification of fish to monitor fishery stocks, diversity, bycatch, and to combat illegal fishing (Marini et al., 2018; Perdigão et al., 2020).

Machine learning techniques have been successfully implemented in niche modeling, prediction of mass mortality events, and the development of non‐linear ecological time‐series models (Crisci et al., 2012; Miller‐Coleman et al., 2012; Recknagel, 2001). Image classification deep learning models show promise in being applied to highly diverse taxa and collections (Borowiec et al., 2021; Norouzzadeh et al., 2018; Sullivan et al., 2018; Wäldchen & Mäder, 2018; Schuettpelz et al., 2017; Weinstein, 2017). Past attempts to identify fish taxa using computer vision have had varying degrees of success across a wide breadth of ichthyological data sets. For example, early attempts by Alsmadi et al. (2010) were able to identify 20 families of marine fish from 610 images with an accuracy of 84%. More recent work improved accuracy to 90% (Alsmadi et al., 2019). Hernández‐Serna and Jiménez‐Segura (2014) used seven museum collections that included both marine and Amazonian freshwater fish (images per collection ranged from 422 to 2392) and obtained accuracies between 72% and 92%. Sun et al. (2016) obtained a species identification accuracy of 77.27% from 9160 AUV images of fish. A study by Qin et al. (2015) was able to identify 23 deep sea fish species with an accuracy of 98% using a substantial number of training images (*n* = 22,370).

In this study, we developed two deep learning computer vision models: one that segments fish pixels from background pixels, and one that classifies images of Amazonian fishes to the genus level. As the first image classifier for ichthyological monitoring in the megadiverse Peruvian Amazon basin, we hope this case study will act as a primer for further development of deep learning models, as tools for conservation stakeholders. Deep learning for taxonomic image classification has proven to be efficient and highly accurate, demonstrating promise for improving participatory monitoring initiatives (Norouzzadeh et al., 2018; Sullivan et al., 2018). Specifically, these tools will enable communities involved in participatory monitoring to fill knowledge gaps and improve data reliability. These models can also provide a basis on which to build new models for other species of conservation concern and public health interest. Our data and pipeline are publicly available, which will enable others to apply these techniques to other taxa.

## 2 METHODS

In July 2018, we sampled freshwater fishes in small white‐water rivers, and black and white‐water streams in seasonally flooded forests of the upper Morona River valley in Achuar native territory, Loreto, Peru. Sites were resampled in November 2018 and November 2019. Fish were identified by specialists with the aid of dichotomous taxonomic keys considering morphological, meristic, and morphometric characteristics. Taxonomic nomenclature follows Fricke et al. (2018). A total of 141 fish species belonging to 89 genera and 29 families across all sites and seasons were identified (M. Ruiz‐Tafur, unpublished data). Captured fish (*n* = 1967) were placed on a 1 cm grid or a neutral background (leaves, hands, ground, etc.) and photographed using a Nikon D3500 camera, prior to preservation. Specimens were deposited in the ichthyology collection at the Instituto de Investigaciones de la Amazonia Peruana (IIAP) in Iquitos, Peru. Due to the limited number of images we had per species, we restricted our analysis to genera (*n* = 33), using a minimum threshold of 20 field images per genus (*n* = 1615). To supplement field images, we incorporated additional images (*n* = 1453) taken of specimens housed at the Smithsonian National Museum of Natural History Department of Vertebrate Zoology, Division of Fishes collection (USNM) using both a Nikon B500 and W100. Fish specimens were photographed on both blank and 1 cm grid backgrounds from multiple angles. In total, our dataset consists of 3068 images prior to processing.

### 2.1 Preprocessing steps

To build a training dataset, we first removed all incidentally taken/non‐fish and unidentified fish images. We then built a U‐Net (Ronneberger et al., 2015) segmentation model to classify pixels in images as fish or background using the methods similar to White et al. (2020). Specifically, we manually masked a subset of images (*n* = 66; 2 images from each genus), using the methods of White et al. (2020), to use as a training set to build a U‐Net. Our generated masks zeroed out (blacked) background pixels, while retaining fish pixels. The model was built on a resnet‐34 architecture pretrained on the ImageNet dataset (Deng et al., 2009). All field and museum images were then masked by our trained U‐Net. Images which were unsuccessfully masked, where no component of the original input image remained within the photo, were removed from the dataset. The remaining images, which had at least some component of the target object with no background, were then subdivided for training and validation of the genus identification model.

### 2.2 Identification model architecture, training, and validation

**FIGURE 1:**
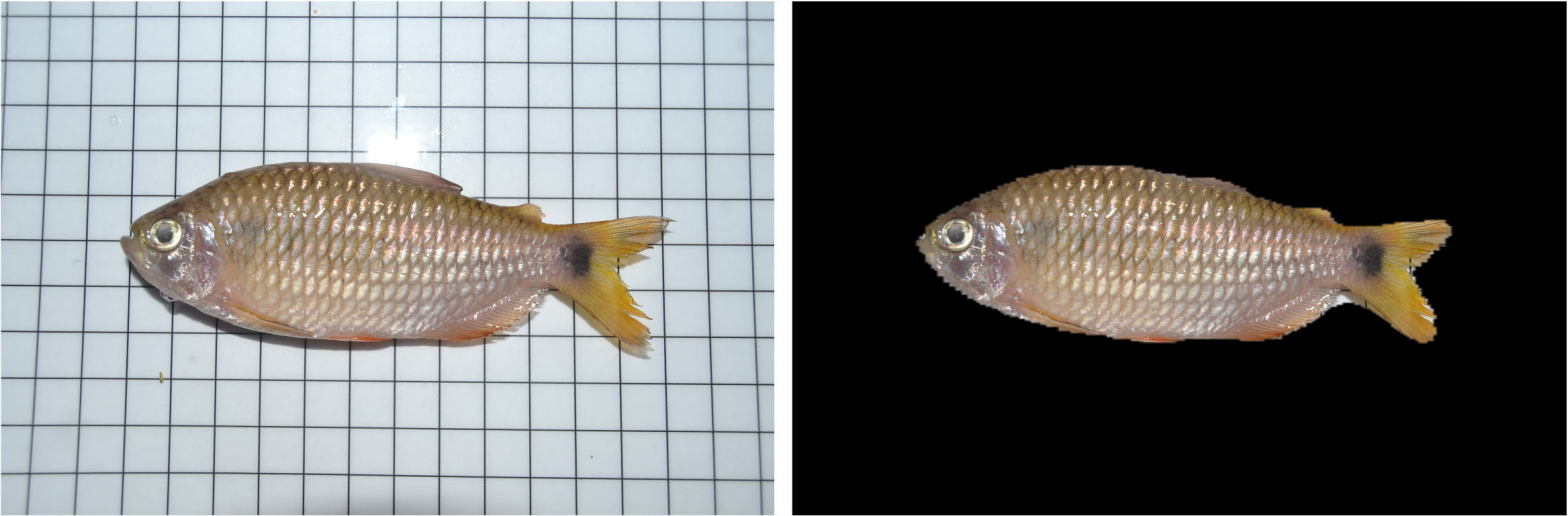
Example of unmasked (left) and masked (right) images of a fish (*Bario steindachneri*).

We trained our image classifier to distinguish between 33 fish genera based on masked images. The classifier was developed using a Nvidia GeForce (V100; 32GB VRAM) GPU implementing the Fast.ai library (Howard & Gugger, 2020) in PyTorch (Paszke et al., 2019). The model was built on a resnet‐101 architecture pretrained on the ImageNet dataset (Deng et al., 2009). To develop our image classifier model, masked images were randomly divided into training (*n* = 2387) and validation (*n* = 596) sets, split 80/20 respectively, to maximize accuracy (Hernández‐Serna & Jiménez‐Segura, 2014). All images were resized by ‘squishing’ them into 300 × 300 pixels. We trained our model over 60 epochs with 1 training session of random transformations making up 6/60 epochs. Ichthyological field work in Peru was approved by the Smithsonian National Zoological Park Institutional Animal Care and Use Committee (NZP‐IACUC Protocol #18‐25) (Figure 1).

## 3 RESULTS

**FIGURE 2:**
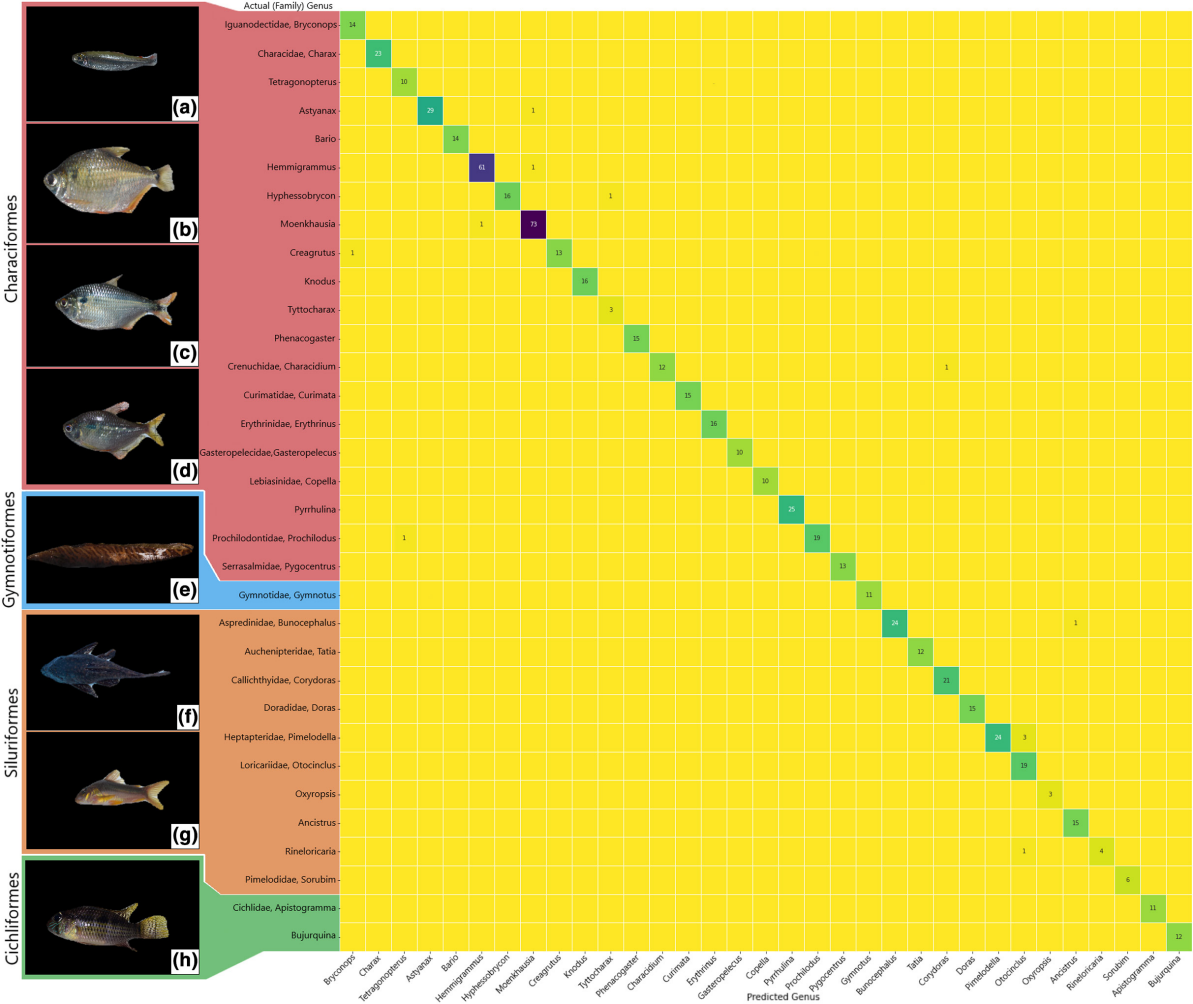
Confusion matrix visualization of computer vision model validation results. The *x*‐axis depicts the genus predicted by the model. The *y*‐axis depicts the actual genus to which the image belongs, organized by taxonomic class, family and genus according to Fricke et al. (2018). Correct identifications are depicted in the left‐to‐right diagonal, with a darker color indicating more correct identifications, and blank yellow squares indicating zeros. Masked image examples on *y*‐axis are as follows: (a) *Bryconops*, (b) *Tetragonopterus*, (c) *Astyanax*, (d) *Moenkhausia*, (e) *Gymnotus*, (f) *Ancistrus*, (g) *Corydoras*, and (h) *Bujurquina*.

The U‐Net masking model was trained over 20 epochs, at which point the training loss and validation loss were minimized. Our U‐Net was able to successfully mask 97.23% (*n* = 2983) of our images. Images which were not successfully masked (*n* = 85) were removed from training and validation. Our Amazonian fish image classifier trained in 50 epochs at which point the training loss and validation loss were minimized. The validation set results, predicted class versus actual class, are summarized in a confusion matrix (Figure 2). Of the 596 validation images, the image classifier predicted 97.99% of them correctly. Accuracy by genus is summarized in Table 1. The range of accuracy by genus ranged from 88.89% to 100%. The models, and associated metadata are available at the Smithsonian Figshare repository (https://doi.org/10.25573/data.17315126). The application for both models is available online (https://sidatasciencelab.github.io/Amazonian_Fish_ML_Classifier/).

**TABLE 1:** Summary of validation set (*n* = 596) results by genus.

Order	Family	Genus	Correct	Incorrect	Total	Accuracy (%)
Characiformes	Iguanodectidae	Bryconops	14	0	14	100
Characiformes	Characidae	Charax	23	0	23	100
Characiformes	Characidae	Tetragonopterus	10	0	10	100
Characiformes	Characidae	Astyanax	29	1	30	96.67
Characiformes	Characidae	Bario	14	0	14	100
Characiformes	Characidae	Hemmigrammus	61	1	62	98.39
Characiformes	Characidae	Hyphessobrycon	16	1	17	94.12
Characiformes	Characidae	Moenkhausia	73	1	74	98.65
Characiformes	Characidae	Creagrutus	13	1	14	92.86
Characiformes	Characidae	Knodus	16	0	16	100
Characiformes	Characidae	Tyttocharax	3	0	3	100
Characiformes	Characidae	Phenacogaster	15	0	15	100
Characiformes	Crenuchidae	Characidium	12	1	13	92.31
Characiformes	Curimatidae	Curimata	15	0	15	100
Characiformes	Erythrinidae	Erythrinus	16	0	16	100
Characiformes	Gasteropelecidae	Gasteropelecus	10	0	10	100
Characiformes	Lebiasinidae	Copella	10	0	10	100
Characiformes	Lebiasinidae	Pyrrhulina	25	0	25	100
Characiformes	Prochilodontidae	Prochilodus	19	1	20	95
Characiformes	Serrasalimidae	Pygocentrus	13	0	13	100
Gymnotiformes	Gymnotidae	Gymnotus	11	0	11	100
Siluriformes	Aspredinidae	Bunocephalus	24	1	25	96
Siluriformes	Auchenipteridae	Tatia	12	0	12	100
Siluriformes	Callichthyidae	Corydoras	21	0	21	100
Siluriformes	Doradidae	Doras	15	0	15	100
Siluriformes	Heptapteridae	Pimelodella	24	3	27	88.89
Siluriformes	Loricariidae	Otocinclus	19	0	19	100
Siluriformes	Loricariidae	Oxyropsis	3	0	3	100
Siluriformes	Loricariidae	Ancistrus	15	0	15	100
Siluriformes	Loricariidae	Rineloricaria	4	1	5	80
Siluriformes	Pimelodidae	Sorubim	6	0	6	100
Cichliformes	Cichlidae	Apistogramma	11	0	11	100
Cichliformes	Cichlidae	Bujurquina	12	0	12	100
		Total	584	12	596	97.99

## 4 DISCUSSION

We were able to efficiently build a state‐of‐the‐art model which can rapidly identify standardized Amazonian fish images to the genus level (*n* = 33) with 97.99% accuracy, in line with the results of other deep learning fish studies implementing image classifiers (Alsmadi et al., 2019; Qin et al., 2015). Of the 12 incorrectly classified images in our validation set, 7 were misclassified outside of their family, while 2 images were misclassified outside of their order. Results demonstrate the importance of image quality, image quantity, and taxonomic specificity to generating image classification models that will prove useful for identifying diverse taxa in remote geographic settings.

In this study, we attempted to control image quality by using masking as a way to standardize images. After visually examining the incorrectly classified images, it was evident that some of them were likely more difficult to classify because of bisection from incidentally masked fish pixels. In short, we believe our masking rendered a few of our images unidentifiable and is arguably an artifact of the data pipeline rather than a source of true error on the image classifier. One way to improve the final classification accuracy is to capture multiple clear images of individual fish to ensure at least one is successfully masked prior to inference for identification. While the original images used in the study were taken at high resolution of varying sizes, they were ultimately resized to just 300 × 300 pixels. The rapid advancement of mobile phone photography (Rasmusson et al., 2004) and availability of mobile phones with cameras capable of capturing images even higher in resolution that those used here (González & Pozo, 2019) will contribute to the ever‐growing quantity of high‐quality image data available to enable generation of even more robust and more accurate models. Standardized protocols for collecting photographic data in both field and museum settings will be key to bolstering future modeling efforts.

The remoteness of the localities sampled as part of this study and the cryptic nature of the species endemic to these sites significantly limited the number of images, we were able to acquire from the field. We combatted the limitation of image quantity by photographing museum specimens available to us. Utilizing a hybrid approach—a combination of field images and digitized museum collection specimens—we were able to double the amount of data available to generate the model. Combining both museum and field collected images to generate a classification model can enable novel insights that may not have been found by building separate museum and field models (Lendemer et al., 2020). The use of multiple data sources, and willingness to make these publicly available, will provide a robust framework for future image classification models with limited available training data.

Most misidentifications in our model involved tetras, small characids that are the dominant fish fauna in Amazonian small rivers and streams (de Oliveira et al., 2009). Historically, species‐rich and closely‐related tetras have been difficult to identify due to cryptic species diversity – where more than one nominal species may be several undescribed species – and the lack of exclusive morphological characters to identify some genera (e.g., *Astyanax* > 170 species and *Hyphessobrycon* > 130 species; Barreto et al., 2017; Escobar‐Camacho et al., 2015; Oliveira et al., 2011). In addition, an estimated 40% of species in the region have yet to be described (e.g., Reis et al., 2016). Thus, species misidentifications due to taxonomically complex groups, such as tetras and other cryptic assemblages, are common problems in manual morphological as well as with genetic identification approaches (e.g., de Santana et al., 2021) and this must be considered when building an image classifier for Amazonian fishes. In short, the output given by an image classification model is only as good as the label given to each class during training. If the target class is not well defined, as it may be in the case of tetras, this may disrupt the classification accuracy of the classification for those genera.

Collection of accurate, reliable biodiversity data is vital for monitoring ecosystem health and co‐benefits for human well‐being. The emergence of new technologies such as mobile applications, wireless sensor networks, augmented/virtual reality and high throughput computing are already advancing scientific research by enabling community scientists to bridge the training gap through instant “expert” verification (Newman et al., 2012). Although previous efforts have applied image classification to citizen science data (Van Horn et al., 2018), none have targeted freshwater fish in such highly biologically and culturally diverse sites as the upper Morona River valley. Given the importance of fish as key indicators of water quality and ecosystem health (Harris, 1995), as well as the dependence of many indigenous Amazonian communities on fish as a reliable source of protein (Swierk & Madigosky, 2014), there is great need for tools that increase the accessibility of taxonomic identification required for accurate monitoring of fishes (Gardiner et al., 2012; Newman et al., 2012). When deployed in the field, our model will empower community‐led initiatives to monitor fish in the Amazon River basin to collect more accurate information and identify ecological trends about this integral source of food and income (Finer et al., 2008). While the model presented here is accurate at identifying fish to the genus level, we expect this to be a first step toward increased digitization and image generation to support training a model at the species level.

Past field efforts have applied image classification to citizen science data taken from the field (Van Horn et al., 2018), but none have targeted freshwater fish in such highly diverse sites as the upper Morona River valley. Image classification models such as the model presented here increase the accessibility of taxonomic identification needed to accurately monitor ecosystem health and natural resources (Gardiner et al., 2012; Newman et al., 2012). In such an incredibly diverse ecosystem, a model accurately identifying fish to the genus level is a first step which will provide motivation for increased digitization efforts to obtain sufficient images for training a model at the species level.

## 5 CONCLUSIONS

We present an application that can be used to rapidly and accurately classify freshwater fish from the upper Morona River valley in the northwest Amazon to genus for scientific research. Although able to classify 33 genera present in the current study area, the model described here provides a solid foundation for future projects. The application, which can be used to classify single images to genus, is accessible to the community online. The model's application to images taken from geographic areas outside of the northwestern Amazon has yet to be explored.

## AUTHOR CONTRIBUTIONS

**Alexander J. Robillard:** Conceptualization (supporting); data curation (equal); formal analysis (lead); investigation (equal); methodology (lead); validation (lead); visualization (lead); writing – original draft (lead); writing – review and editing (equal). **Michael G. Trizna:** Conceptualization (supporting); data curation (supporting); formal analysis (supporting); funding acquisition (supporting); investigation (supporting); methodology (supporting); resources (supporting); software (equal); supervision (equal); validation (equal); visualization (equal); writing – review and editing (supporting). **Morgan Ruiz‐Tafur:** Data curation (supporting); investigation (supporting); methodology (supporting); resources (supporting); writing – original draft (supporting); writing – review and editing (supporting). **Edgard Leonardo Dávila Panduro:** Data curation (supporting); investigation (supporting); methodology (supporting); resources (supporting); writing – original draft (supporting); writing – review and editing (supporting). **C. David de Santana:** Resources (supporting); validation (supporting); visualization (supporting); writing – original draft (supporting); writing – review and editing (supporting). **Alexander E. White:** Formal analysis (supporting); investigation (supporting); methodology (supporting); software (supporting); supervision (supporting); validation (supporting); writing – review and editing (supporting). **Rebecca B. Dikow:** Conceptualization (equal); data curation (supporting); formal analysis (equal); funding acquisition (equal); investigation (equal); methodology (equal); project administration (equal); resources (lead); software (lead); supervision (lead); validation (equal); visualization (supporting); writing – original draft (supporting); writing – review and editing (supporting). **Jessica L. Deichmann:** Conceptualization (lead); data curation (supporting); formal analysis (supporting); funding acquisition (lead); investigation (supporting); methodology (supporting); project administration (lead); resources (lead); software (equal); supervision (lead); validation (supporting); visualization (supporting); writing – original draft (equal); writing – review and editing (equal).

## CONFLICT OF INTEREST STATEMENT

The authors have no conflicts of interest.

## Data Availability

Image masks, masked training data data, associated metadata used for model generation, and both models are available on the Smithsonian Figshare (https://doi.org/10.25573/data.c.5761097). The web‐application is available here (https://sidatasciencelab.github.io/Amazonian_Fish_ML_Classifier/), and on Github (https://github.com/MikeTrizna/streamlit_fish_masking).
